# Nano-strategies for Targeting Tumor-Associated Macrophages in Cancer immunotherapy

**DOI:** 10.7150/jca.108194

**Published:** 2025-03-31

**Authors:** Qian Li, Jingwei Xu, Runjia Hua, Hanye Xu, Yongyou Wu, Xiaju Cheng

**Affiliations:** 1 Department of General Surgery, The Second Affiliated Hospital of Soochow University, Suzhou 215004, P. R. China.; 2 Key Laboratory of Radiation Medicine and Protection, School for Radiological and Interdisciplinary Sciences (RAD-X), Collaborative Innovation Center of Radiation Medicine of Jiangsu Higher Education Institutions, Soochow University, Suzhou 215123, P. R. China.; 3 Department of Thoralic Surgery, Suzhou Municipal Hospital Institution, Suzhou 215000, P. R. China.; 4 Department of Thoralic Surgery, Dushu Lake Hospital Affiliated to Soochow University, Suzhou, 215123, P. R. China.; Abstract:

**Keywords:** Cancer immunotherapy, Tumor-associated macrophages, Nanodrugs, M1 polarization, Signaling pathways

## Abstract

Tumor-associated macrophages (TAMs) are one type of the most abundant immune cells within tumor, resulting in immunosuppresive tumor microenvironment and tumor resistance to immunotherapy. Thus, targeting TAMs is a promising therapeutic strategy for boosting cancer immunotherapy. This study provides an overview of current therapeutic strategies targeting TAMs, which focus on blocking the recruitment of TAMs by tumors, regulating the polarization of TAMs, and directly eliminating TAMs using various nanodrugs, especially with a new categorization based on the specific signaling pathways, such as NF-κB, HIF-1α, ROS, STAT, JNK, PI3K, and Notch involved in their regulatory mechanism. The latest developments of nanodrugs modulating these pathways are discussed in determining the polarization of TAMs and their role in the tumor microenvironment. Despite the challenges in clinical translation and the complexity of nanodrug synthesis, the potential of nanodrugs in enhancing the effectiveness of cancer immunotherapy is worthy of expecting.

## Introduction

**Figure 1:**
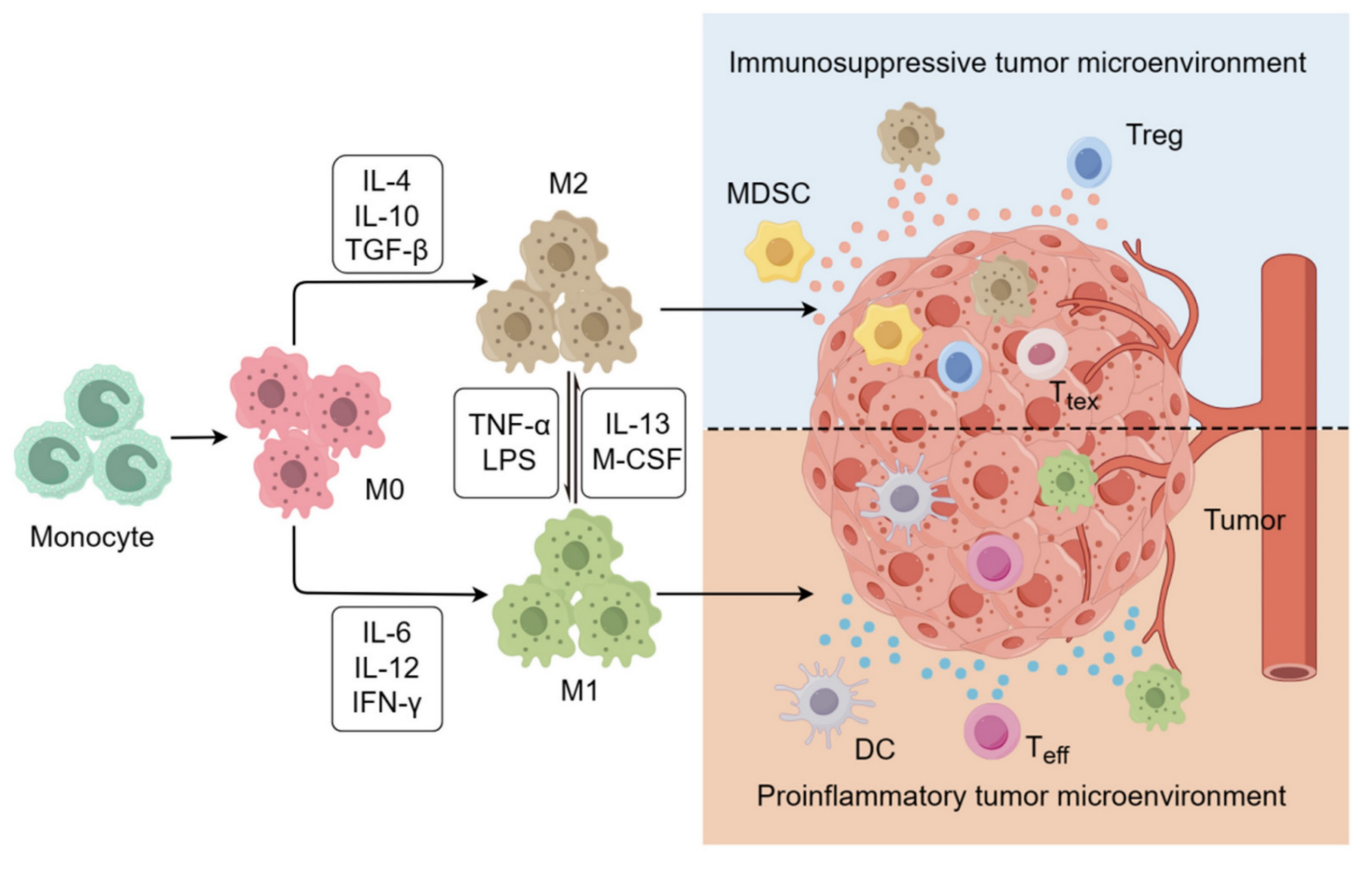
The polarization of macrophages. M0 macrophages can differentiate into either M1 or M2 macrophages when stimulated by different cytokines. M1 macro-phages are known for their involvement in inflammatory responses and have both proinflammatory and anti-tumor effects. In contrast, M2 macrophages are commonly found in the immunosuppresive microenvironment, where they exert an anti-inflammatory effect and contribute to tumor growth and metastasis.

Cancer immunotherapy can be designed to harness the innate and adaptive immune systems to attack tumor cells, ultimately improving the survival outcomes of cancer patients, particularly those in advanced stages. This approach has emerged as a focal point in both basic and clinical cancer research 1. Although clinical trials have shown excellent results, several key challenges still need to be addressed, including low response rates, durability of response, immune-related adverse events, and atypical clinical responses 2. Recent investigations have highlighted the pivotal role of tumor-associated macrophages (TAMs) in mediating resistance to immunotherapy, given their prominence as key immunosuppressive cells within the tumor microenviron-ment (TME) 3. TAMs derived from circulating monocytes and recruited to the tumor site dynamically shape the TME, fostering conditions conducive to tumor growth and progression 4. Macrophages are divided into two main subsets based on their phenotype and function: classically activated M1 macrophages and alternatively activated M2 macrophages (Figure 1). M1 macrophages typically contribute to anti-tumor immunity by engaging in antigen presentation and producing proinflammatory cytokines like interleukin-6 (IL-6), interleukin-12 (IL-12), and interleukin-1 (IL-1). M2 macro-phages promote tumor growth by secreting a series of anti-inflammatory cytokines such as interleukin-4 (IL-4), interleukin-10 (IL-10), and transforming growth factor beta (TGF-β) 5. Macrophages infiltrating the tumor site account for approximately 50% of immune cells in the TME 6. Within the TME, the polarization of tumor infiltrating TAMs can be influenced by various factors, ultimately adopting an M2-like phenotype owing to conditions such as hypoxia, oxidative stress, and lactate accumulation 7. These M2-like TAMs secrete differential immunosuppressive cytokines that can enhance the recruitment of other immunosuppressive cells, such as regulatory T cells (Tregs) and myeloid-derived suppressor cells (MDSCs), while also contributing to the conversion of effector T cells (T_eff_) to exhausted T cells (T_tex_), ultimately fueling tumor proliferation, angiogenesis, and metastasis 8, 9. This close association with tumor prognosis highlights the importance of targeting TAMs to enhance the effectiveness of the immune system in combating tumors 10, 11.

However, traditional drugs targeting TAMs have some limitations, including inadequate specificity, notable side effects, and potential for drug resistance 12. In this context, nanodrugs may partially address these issues by improving drug stability, solubility, and bioavailability 13, 14. Additionally, nanodrugs can be rationally designed to possess targeted delivery capabilities through passive or active targeting, thereby minimizing the off-target effects. Numerous novel nanodrugs have been developed to enhance cancer immunotherapy by targeting TAMs, showing promising preclinical and clinical results 15, 16. This paper reviews nanodrugs that target TAMs and discusses the underlying challenges in their development.

## Therapeutic strategies for targeting TAMs

**Figure 2:**
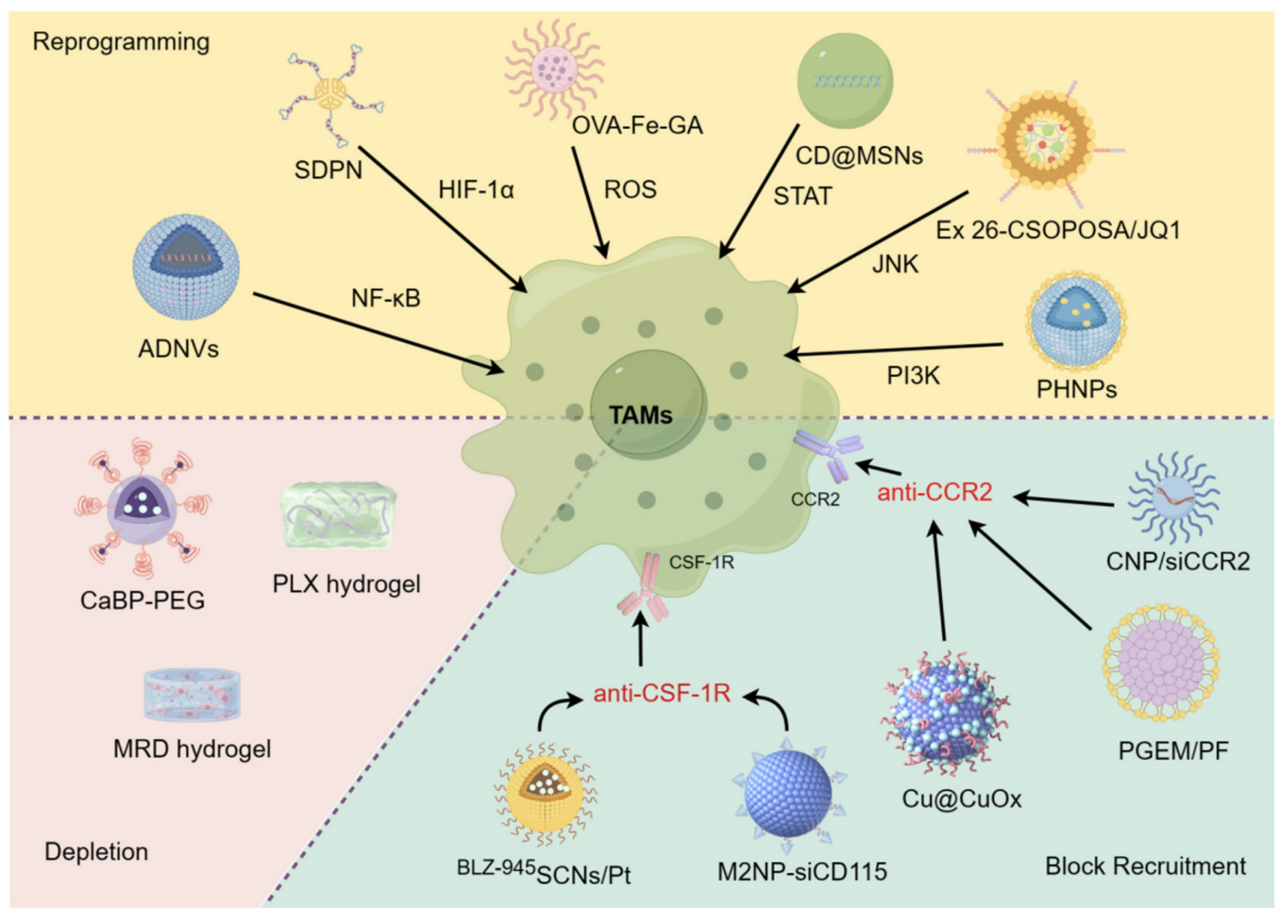
Current therapeutic strategies for targeting TAMs with nanodrugs. Through blocking tumor recruitment of TAMs, depleting/suppressing TAMs, and reprogramming TAMs, the goal is to reshape the TME and enhance the immune response against cancer. Nanodrugs have the potential to precisely deliver and release therapeutic agents, offering a promising approach to enhance the effectiveness of TAM-targeting immunotherapies while minimizing side effects.

TAMs play a critical role in promoting tumor growth and resistance to treatment in various cancer types. These discoveries lay a strong groundwork for targeting these cells and their precursors to enhance patient outcomes 17. Current therapeutic strategies for targeting TAMs using nanodrugs can be broadly divided into three approaches: blocking TAMs recruitment to the TME, depleting and/or suppressing TAMs, and reprogramming TAMs polarization to the M1 phenotype (**Figure 2**) 18-20.

### Blocking tumor recruitment of TAMs

Monocytes/macrophages are recruited to the TME by various factors secreted by tumor cells and tumor stromal cells. Once infiltrated, these monocytes/macrophages are educated by the TME and differentiate into TAMs, serving as a continuous supply of TAMs. Therefore, targeting key factors involved in monocyte/macrophage recruitment is crucial. Among these factors, colony stimulating factor 1(CSF-1) plays a crucial role in the recruitment of monocytes to the TME 21-24. CSF-1 interacts with the colony stimulating factor 1 receptor (CSF-1R) on monocytes, leading to their polarization into TAMs 25-27. Monocyte chemoattractants, such as C-C motif chemokine ligand 2 (CCL2) and its homologous receptor C-C chemokine receptor type 2 (CCR2), are targeted for the treatment of solid tumors and hematological malignancies using monoclonal antibodies and receptor antagonists. Many antibodies and small molecule drugs targeting the CCL2/CCR2 axis have entered clinical trial stages. However, most of them have not achieved the desired efficacy, such as carumab 28. Nanomaterials offer a potential solution for enhancing drug efficacy through targeted delivery and sustained release.

Zhang* et al.* developed CCR2-targeted small copper nanoparticles (Cu@CuOx) that can inhibit TAMs recruitment and deliver the chemotherapeutic drug gemcitabine to treat pancreatic ductal adenocarcinoma (PDAC). By covalently conjugating the CCR2-targeting peptide ECL1i and gemcitabine to nanoparticles, Cu@CuOx nanoparticles ingeniously achieves precise targeting of tumor cells and effective drug delivery. These nanoparticles also enabled PET imaging owing to their ^64^Cu radiolabeling. Systemic administration of gemcitabine-loaded Cu@CuOx in a PDAC xenograft mouse model significantly reduced tumor growth and extended survival 29. Möckel* et al.* studied the effect of the surface charge of polymer nanoparticles on the biodistribution of monocytes for targeted delivery. They discovered that positively charged nanoparticles accumulated better in monocytes, and after carrying siCCR2 (CNP/siCCR2), they could significantly improve anti-tumor outcomes by modifying the TME in a mouse breast cancer model 30. Wan* et al*. incorporated the CCR2 antagonist PF-6309 (PF) into a polymeric micelle system based on gemcitabine (PGEM). The PGEM/PF formulation effectively reversed CCL2/CCL7-mediated immune suppression and significantly induced potent anti-tumor immunity to reduce the pancreatic tumor burden. This study paves a new avenue for the rational design of nanomedicine based on bioinformatics and computational modeling 31. In addition, Shen* et al.* created a dual-targeting immunostimulatory nanocarrier (^BLZ-945^SCNs/Pt) for chemoimmunotherapy that simultaneously addresses TAMs and tumor cells. This nanocarrier was designed to release a platinum (Pt) prodrug and a CSF-1R inhibitor (BLZ-945) into the prevascular areas of tumors. The ^BLZ-945^SCNs/Pt undergo supersensitive structural collapse in the prevascular regions of tumor tissues, simultaneously releasing Pt-prodrug-conjugated small particles and BLZ-945. The released BLZ-945 can be preferentially taken up by TAMs, leading to the depletion of TAMs from tumor tissues. Meanwhile, the small particles carrying Pt-prodrug can penetrate deeply into the tumor and intracellularly specific release drug to kill more cancer cells. This pH-sensitive co-delivery nanocarrier not only induces apoptosis of tumor cells but also modulates the tumor immune microenvironment, ultimately enhancing the antitumor effect of CD8+ cytotoxic T cells through the depletion of TAMs 32. Taken together, the interception of TAM recruitment represents a pivotal strategy for altering the polarization of TAMs toward an immunesuppressive phenotype, offering significant promise for future therapeutic development.

### Depleting and/or suppressing TAMs

Multiple preclinical and clinical studies have shown that reducing the number of TAMs can inhibit tumor progression and improve treatment outcomes 33-38. Because TAMs are primarily derived from circulating monocytes, depleting monocytes is also another way to reduce the number of TAMs. Some targeted drugs have been able to deplete TAM populations or inhibit TAM activity to some extent. Nevertheless, the primary obstacles that remain are the lack of specificity, high toxicity, and limited efficacy 39.

Recently, Zhang* et al.* developed an ATP-supersensitive nanogel (B^BLZ-945^@PAC-PTX) that sequentially eliminates these barriers. Upon reaching the tumor, it collapses in response to ATP, releasing B^BLZ-945^ to deplete TAMs. The shrunk nanogel (PAC-PTX) penetrates deeper, blocking CXCR4 to reduce immunosuppressive cells and internalizing into tumor cells for killing and T cell priming. This "dominolike" strategy enhances immune cell infiltration and boosts immune response, providing a high-responsive chemoimmunotherapy option 40. Zhou* et al.* developed an engineered biomimetic nano-red blood cell system, denoted as V(Hb)@DOX, which targets the depletion of M2-type TAMs, alleviates tumor hypoxia, and holds significant potential for reprogramming the TME and enhancing the efficacy of chemoimmunotherapy 41. Similarly, Li* et al*. developed a biocompatible alginate-based hydrogel designed for localized pexidartinib (PLX) release to effectively deplete TAMs. This strategy not only promotes T cell infiltration, but also synergies with platelet activation post-surgery to enhance the release of aPD-1, thereby boosting the immunotherapeutic efficacy against tumor recurrence 42. Additionally, the clinical drug bisphosphonates, known for their ability to deplete TAMs 43, were utilized by Liu* et al.* to prepare CaBP-PEG nanoparticles via a mineralization method using a poly(polyethylene glycol) (PEG) coating. CaBP-PEG nanoparticles composed of Ca^2+^ and bisphosphonates are highly biocompatible and multifunctional. Through innovative chelator-free radiolabeling chemistry, the authors successfully developed CaBP(^99m^Tc)-PEG and CaBP(^32^P)-PEG for SPECT imaging guided enhanced cancer radioisotope therapy, and demonstrating its potential and clinical translation value in cancer treatment 44. Nevertheless, while reducing TAMs can weaken their role in promoting tumor growth, the immunosuppressive environment of TME frequently hinders the success of treatment approaches. In this regard, rather than eliminating TAMs, reprogramming them could be a more beneficial approach to counteract the immunosuppressive TME and improve the effectiveness of drug therapies.

### Reprogramming TAMs into M1-type macrophages

**Figure 3:**
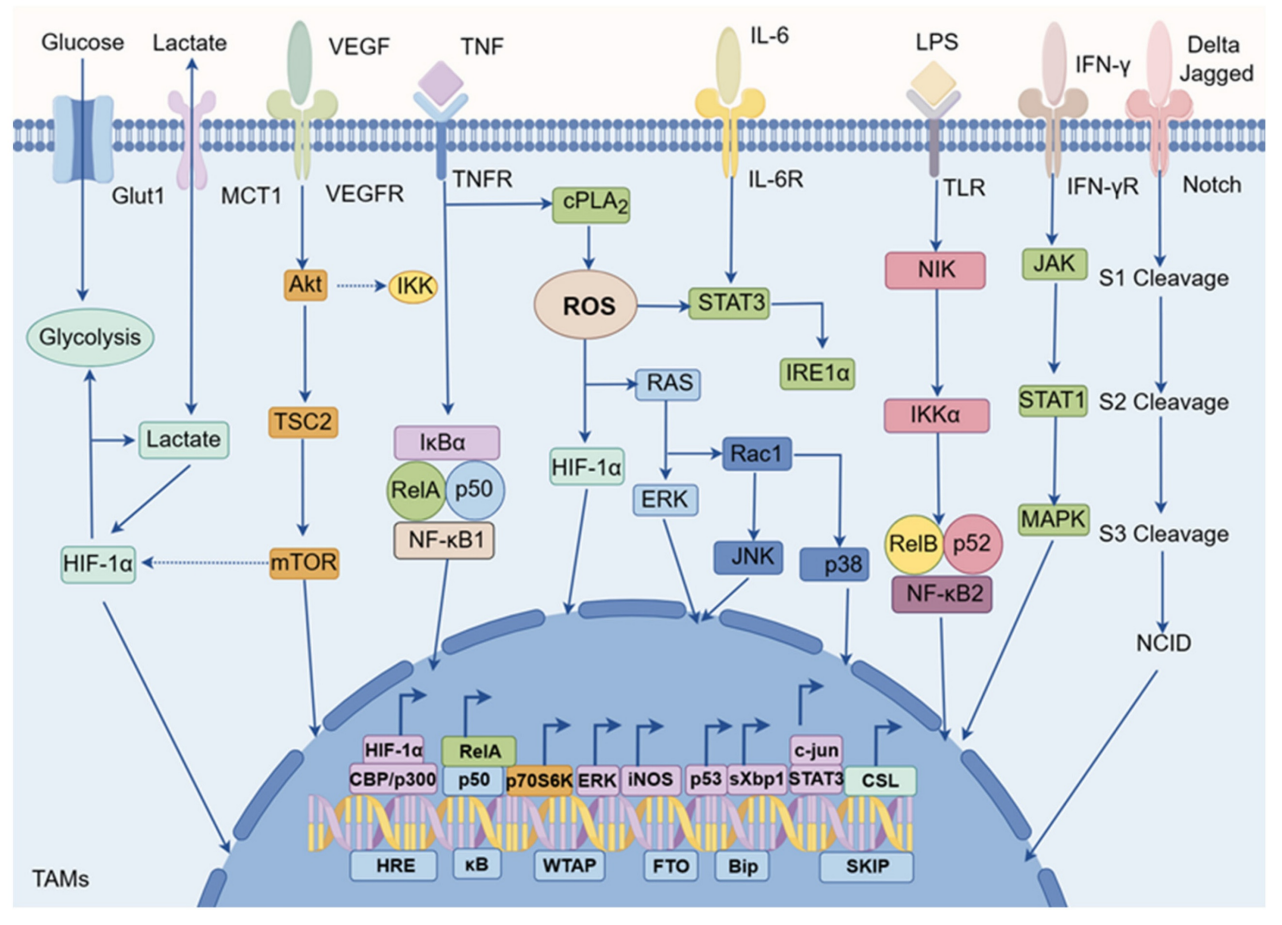
Cellular signaling networks in TAMs. The cellular signaling networks within TAMs are vital for regulating their polarization. Signaling pathways such as NF-κB, HIF-1α, ROS, STAT, JNK, PI3K, and Notch play a critical role in determining the direction of TAM polarization. These pathways can function independently or interact with each other to influence TAM polarization. Consequently, they present potential targets for nanodrugs in cancer immunotherapy focused on TAMs.

Currently, there is significant interest in reprogramming TAMs by activating specific signaling pathways associated with macrophages, which can induce polarization towards an anti-tumor M1 phenotype. In TAMs, various signaling pathways are involved in the regulation of TAM polarization, such as nuclear factor-κB (NF-κB), hypoxia inducible factor-1 alpha (HIF-1α), reactive oxygen species (ROS), signal transducer and activator of transcription (STAT), c-Jun NH2-terminal kinase (JNK), phosphatidylinositol 3-kinase (PI3K), and Notch etc (Figure 3) 45, 46. For instance, activation of the NF-κB signaling pathway typically promotes TAMs polarization towards the M1 direction, while other pathways such as HIF-1α might be closely associated with M2-like TAMs 47. Therefore, targeting these signaling pathways in TAMs holds promise for phenotypic reprogramming 48.

Preclinical studies have shown that nanomaterial-mediated reprogramming of TAMs exhibits significant anti-tumor efficacy 49-51. These nanomaterials not only modify the phenotype of TAMs but also activate T cells and other immune cells, thereby bolstering the overall immune system's assault on the tumor. The success of this approach hinges on the meticulous design of nanomaterials, encompassing factors such as size, shape, surface characteristics, and payload selection, all of which impact their distribution, targeting, and biological effects in the body. Subsequently, our focus shifts to a review of nanodrugs targeting specific signaling pathways involved in TAM polarization (**Table 1**).

#### NF-κB pathway

The NF-κB signaling pathway is a crucial pathway involved in immunity, stress response, cell apoptosis, and differentiation, and it plays a key role in the development of various diseases, including inflammatory infections, autoimmune and metabolic disorders, and cancer 52, 53. This signaling pathway also exists in TAMs and is crucial in determining their polarization. Inhibition of NF-κB signaling pathway-mediated immune suppression related to M2 macrophages can hinder the metastasis of breast cancer cell 54. Furthermore, the involvement of the NF-κB signaling pathway can be observed in almost all fundamental characteristics of cancer 55.

**Figure 4:**
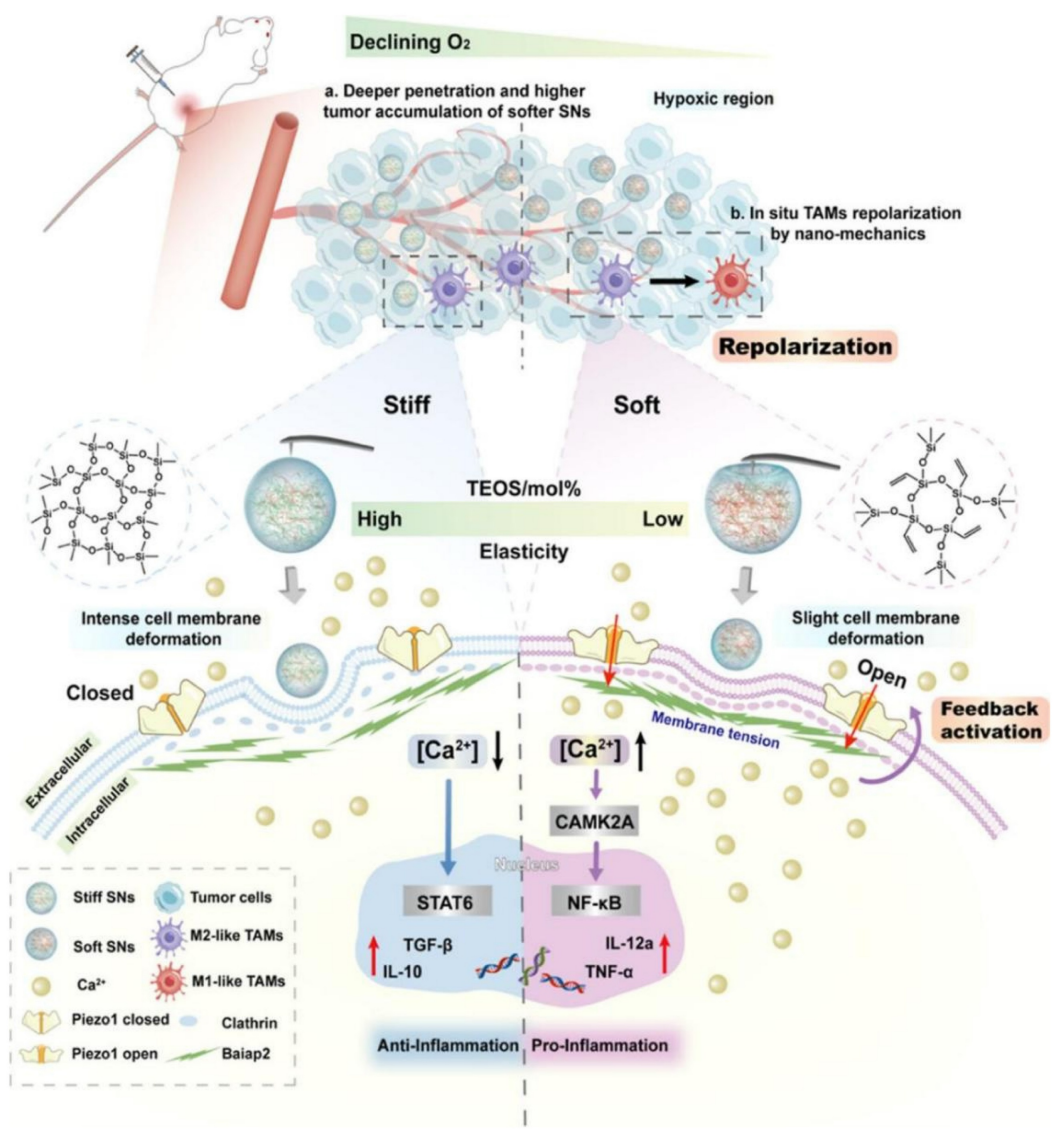
Schematic illustration of the interaction between nanobiointerfaces and TAM repolarization in situ by SNs elasticity. The stiff and soft SNs interact with the macrophage cell membrane, and the soft SNs activate Piezo1 through slight plasma membrane deformation, further inducing Ca^2+^ influx and activating the NF-κB pathway. The soft SNs can penetrate the intratumoral hypoxic regions and reprogram TAMs in situ (Copyright © 2024 American Chemical Society).

Nanodrugs employ two primary strategies to modulate the NF-κB signaling pathway in TAMs: 1) acting as nanocarriers to encapsulate NF-κB pathway inhibitors, which allows for targeted regulation of TAMs, and 2) directly influencing the NF-κB signaling pathway through the intrinsic properties of the nanodrugs themselves 56, 57. Liu* et al.* isolated Artemisia-derived nanovesicles (ADNVs) from Artemisia annua, which are naturally occurring exosome-like nanoparticles that can carry plant-derived mitochondrial DNA (mtDNA). This mtDNA within ADNVs activates the NF-κB pathway, leading to the reprogramming of TAMs into anti-tumor M1 phenotypes, thus offering a novel approach to cancer therapy 58. Chen* et al.* designed an ingenious in situ self-assembled nanomicelle dissolving microneedle (DMN) patch for intralesional delivery of immunogenic cell death-inducer (IR780) and autophagy inhibitor (chloroquine, CQ) co-encapsulated micelles (C/I-Mil), which can act as an im-mune modulator to remodel TAMs toward the M1 phenotype by activating NF-κB 59. Fredrich* et al.* developed a kind of glycated nanoparticles targeted to myeloid cells, containing three therapeutic payloads to modulate the interaction of TAMs and tumor cells, which targeted the toll-like receptor (TLR), NF-κB, and Janus Kinase (JAK) signaling pathways to drive TAMs towards a distinct anti-tumor phenotype characterized by increased IL-12 production and T cell activation 60. In addition, Yang* et al.* explored the biological effects of elastic silica nanoparticles (SNs) with variational elasticity Young's moduli ranging from 81 to 837 MPa on macrophages, showing that soft SNs can repolarize TAMs to M1. The elasticity of SNs affects cell endocytosis, membrane tension, curvature protein Baiap2, and cytoskeleton. Moreover, they can activate the mechanosensitive protein Piezo1 leading to calcium ion influx, NF-κB pathway activation, and inflammatory response (Figure 4). The approach of directly targeting macrophages using the material itself is relatively rare. As a framework, this kind of material can further enhance the targeting and repolarization of TAMs in addition to drug delivery 61. In another study, Cao* et al.* identified ginsenoside nanopar-ticles (GDNPs), a new class of extracellular vesicle-like particles from ginseng, which served as an immunomodulatory agent capable of altering macrophage polarization from the M2 to M1 phenotype. Their study showed that GDNPs, through components such as ceramide lipids and proteins, potentially activate Toll-like receptor 4 (TLR4), leading to a significant reduction in melanoma growth in mice and an increase in M1 macrophages within tumor tissues 62. Zhao* et al.* investigated the anticancer potential of macrophages activated by combining ferumoxytol (FMT) with TLR3 agonist poly(I:C) or poly(I:C)-functionalized FMT nanoparticles (FP-NPs). They demonstrated that FP-NPs did not affect the viability of B16F10 cells but selectively inhibited tumor growth by shifting TAMs to the M1 phenotype via the NF-κB signaling pathway 63. Furthermore, *Shi et al.* successfully reprogrammed TAMs to an anti-tumor M1 phenotype using a photo-generation technology based on mannose-modified PLGA, in which co-encapsulated ICG and TiO_2_ with NH_4_HCO_3_ (denoted as MAN-PLGA-N). MAN-PLGA-N modulates the NF-κB signaling pathway in TAMs. This approach demonstrated superior efficiency, surpassing that of LPS stimulation, representing significant progress in the development of novel cancer therapeutic approaches 64.

These studies highlight the innovative strategies employed by nanodrugs to modulate the NF-κB signaling pathway in TAMs, offering new insights into cancer immunotherapy. The use of natural and synthetic nanomaterials to directly target and repolarize TAMs represents a significant advancement, which not only enhances the delivery of therapeutic agents but also directly influences the tumor microenvironment. However, the translation of these strategies to clinical settings will require further research to address issues such as biocompatibility, toxicity, and the long-term effects of nanomaterials in the body. Additionally, the development of more sophisticated targeting ligands and delivery systems will be crucial to ensure the specificity and effectiveness of these nanodrugs in human trials.

#### HIF-1α pathway

Studies have shown that the expression of HIF-1α is primarily induced by the complex tumor microenvironment, including conditions such as intratumoral hypoxia and elevated lactate levels, which subsequently trigger a series of responses 55, 65. The increased expression of HIF-1α contributes to the recruitment of TAMs and the polarization of M2-like TAMs, resulting in enhanced tumor proliferation, migration, invasion, angiogenesis, and drug resistance 66. HIF-1α accumulates in the nucleus and binds to short DNA sequences called hypoxia response elements (HREs), which are located near oxygen-sensitive genes such as vascular endothelial growth factor (VEGF) 67. The expression of these genes demonstrates why macrophages can express VEGF in avascular and hypoxic areas, explaining the mechanism behind angiogenesis in a low-oxygen, low-pH microenvironment 68.

Recently, various nanodrugs have been developed to regulate macrophage polarization in the TME by targeting HIF-1α. Gu* et al*. developed a hybrid nanoparticle called sophorolipid-associated membrane-biomimetic choline phos-phate-poly(lactic-co-glycolic) acid (SDPN). These nanoparticles are associated with sophorolipids, which provide colloidal stability and rapid diffusion in mucus, resulting in improved endocytosis driven by the interaction between dipalmitoyl choline phosphate and phosphatidyl choline, as well as optimized membrane fluidity and rigidity of SDPN. When loaded with luteolin and silibinin, SDPN regulated the conversion of M2 TAMs into the M1 phenotype and reduced the proportion of the M2 phenotype through co-action on STAT3 and HIF-1α 69. Additionally, Shobaki* et al.* employed a lipid nanoparticle (LNP) formulation to target and deliver siSTAT3/siHIF-1α to TAMs. Using the optimized siRNA-loaded CL4H6-LNPs to target TAMs, they achieved an an-titumor therapeutic effect in a human tumor xenograft model through the silencing of signal STAT3 and HIF-1α, thereby increasing the number of M1-type macrophages 70. Visibly, these nanodrugs can modulate the HIF-1α pathway to repolarize TAMs from pro-tumor M2 to anti-tumor M1 phenotypes, thus boosting the immune response against cancer. Sophisticated nanoparticle formulations like SDPN and LNPs enhance drug delivery and efficacy. These strategies offer new cancer treatment avenues and underscore the importance of targeting the tumor microenvironment to amplify immunotherapy effectiveness.

#### ROS pathway

ROS are typically generated as byproducts of oxygen consumption and cellular metabolism through the partial reduction of oxygen 71. Under oxidative stress conditions, excessive ROS can damage cellular proteins, lipids, and DNA, leading to fatal cell injuries and various pathologies, such as aging, cancer, neuro-degenerative diseases, cardiovascular diseases, and diabetes 72, 73. While ROS are often viewed as harmful to cells, they also play crucial roles in regulating signal transduction pathways 74, 75 and gene expression 76. When addressing hypoxia within TME, the presence of oxygen can trigger the generation of free radicals via the ROS signaling pathway, influencing the polarization and function of TAMs. Notably, ROS are interconnected with several signaling pathways such as JNK, STAT3, HIF-1α, and PI3K 77, 78. For instance, in non-small cell lung cancer (NSCLC), tumor-derived nicotinamide adenine dinucleotide phosphate (NADPH) oxidase recruits M2-TAMs through ROS/PI3K-dependent production of various cytokines, thereby facilitating the growth of NSCLC cells 79.

Numerous nanodrugs have been developed to target ROS in biomedicine field. Most of nanodrugs are developed with the aim of addressing hypoxia in the TME, leading to the activation of the ROS signaling pathway 80. Inspired by the role of NADPH oxi-dase 2 (NOX2), Zhang* et al.* developed a novel nanosystem named OVA-Fe-GA (OFG), which integrates ovalbumin (OVA) and a network composed of Fe-gallic acid (GA) to emulate the ROS generation process of the NOX2 enzyme (O_2_ to O_2_^•-^, H_2_O_2_ and •OH). OFG to reprograme TAMs towards the M1 phenotype and significantly enhances their antigen cross-presentation in mice carrying B16-OVA tumors 81. Li* et al.* developed a nanolipid-based delivery system for C6-ceramide (LipC6), which reduced the number of TAMs and their ROS production. LipC6 was found to induce the differentiation of TAMs into the M1 phenotype, leading to a decrease in immunosuppression and enhancement of CD8+ T activity 82. Moreover, during a study on the molecular mechanisms of Fe_3_O_4_ nanoparticle-induced ferroptosis in macrophages and polarization to-wards the M1 phenotype, Shi* et al*. discovered that both macrophage ferroptosis and TAMs polarization are characterized by high levels of ROS expression 83. Recently, Yang* et al.* developed an oxygen-consumption-responsive nano-ultrasonic contrast agent, Pt(IV)/CQ/PFH NPs-DPPA-1, based on the ROS signaling pathway. This agent reprograms the metabolism of immature dendritic cells (iDCs) and TAMs to enhance the ratio of mature dendritic cells (mDCs) and proinflammatory M1 cells 84. Taken together, these nanodrugs can achieve precise treatment of tumors and regulation of the TME by targeting the ROS signaling pathway in TAMs. Given the complexity of ROS, further research and development will contribute to optimizing the design and utilization of nanodrugs to improve ROS specificity and maximize their potential in ROS-regulated therapies.

#### STAT pathway

STAT proteins belong to a cytoplasmic transcription factor family characterized by a conserved overall structure and modular domains 85. Among these, STAT3 and STAT1 play pivotal roles in macrophage polarization. The expression of STAT1 protein is primarily induced by IFN-γ activation, as it acts as a downstream effector of IFN-γ signaling 86. In TAMs, IFN-γ has been shown to enhance the M1/M2 ratio by down-regulating miR-3473b 87. On the other hand, STAT3 is implicated in various biological processes, including cell proliferation, survival, differentiation, and angiogenesis 88, 89. However, the STAT3 signaling pathway tends to promote macrophage polarization towards the M2 phenotype 90. Recent research has highlighted that STAT3 activation contributes to tumor progression by upregulating protease expression in TAMs 91. Subsequent experiments revealed that conditioned medium from tumor cells triggers inositol-requiring enzyme-1 (IRE-1) signaling, leading to TAM polarization through concurrent activation of STAT3 and production of synaptotagmin-binding protein 1 (sXBP1) 92, 93. Consequently, the activation of the STAT3 signaling pathway is intricately linked to TAM behavior.

**Figure 5:**
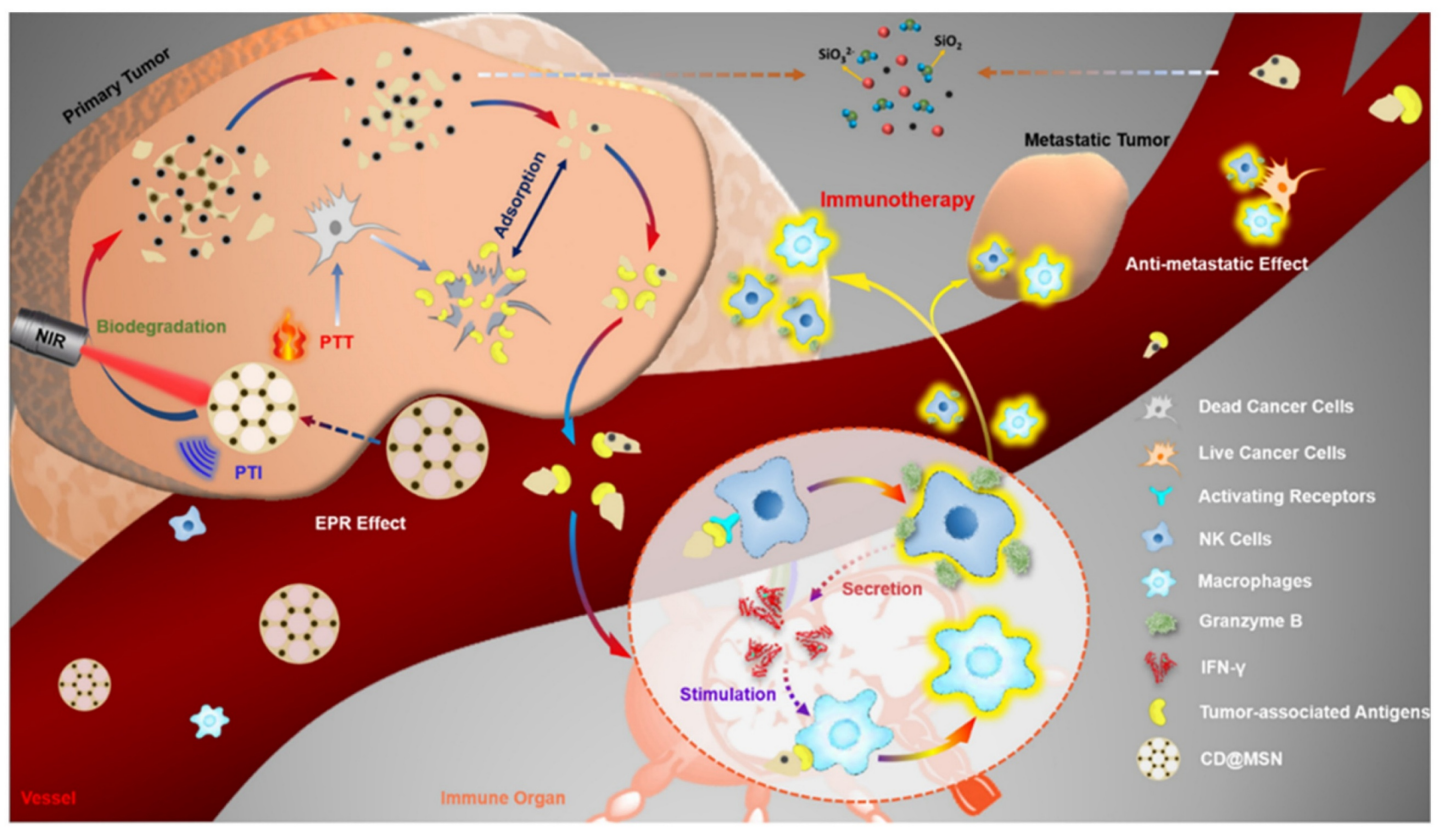
Schematic diagram of in vivo delivery process of framework swelling-triggered biodegradable CD@MSN and its application for photothermal imaging-guided tumor-targeted PTT cooperated with debris-mediated immunotherapy against tumor metastasis (Copyright © 2019 American Chemical Society).

Nanodrugs have emerged as promising tools for regulating the polarization state of TAMs in the TME through modulation of the STAT signaling pathway. Qian* et al*. employed a hydrogen-bonding/electrostatically assisted co-assembly strategy to uniformly integrate carbon nanodots (CDs) into a mesoporous silica framework (CD@MSNs). Combined with photothermal therapy, CD@MSNs can stimulate NK cells to secrete IFN-γ, which might activate STAT1, promote the polarization of TAMs towards the M1 phenotype, and achieve immune-mediated suppression of tumor metastasis (Figure 5). Although CD@MSNs does not directly bind to targets on tumor-associated macrophages, it instead promotes the secretion of anti-tumor cytokines by other immune cells, thereby achieving the effect of targeting macrophages. This approach not only targets TAMs effectively but also leverages the body's immune system to fight cancer, providing a more comprehensive and robust treatment strategy. 94. Moreover, the unique (ginsenoside Rg3- paclitaxel- Liposomes) Rg3-PTX-LPs, loaded with paclitaxel (PTX), were prepared using the thin-film hydra-tion method described by Zhu* et al.* The TME remodeling mechanism of Rg3-PTX-LPs included the suppression of IL-6/STAT3/p-STAT3 pathway activation to polarize pro-tumor M2 macrophages into an anti-tumor M1 phenotype, inhibition of MDSCs, reduction of tumor-associated fibroblasts (TAFs), and collagen fibers in the TME, ultimately promoting tumor cell apoptosis 95. Guo* et a*l. engineered two-dimensional carbon-based nanomaterials, specifically graphdiyne oxide (GDYO) nanosheets, to interact with an intracellular protein corona comprising STAT3. This interaction induces the expression of an anti-tumor phenotype in TAMs 96. Furthermore, based on this signaling pathway, Xu* et al*. developed a nano-immuno-synergist (DPAM@OA) to restore inactivated macrophages in the ma-lignant glioma microenvironment 97. Therefore, these findings indicate that nanodrugs can induce the conversion of M2-like TAMs to M1 macrophages by regulating the STAT1/3 signaling pathway, thereby promoting anti-tumor immune responses and inhibiting the growth and metastasis of tumor cells.

#### JNK pathway

The JNK signaling pathway is a subgroup of the mitogen-activated protein kinase (MAPK) family, which is primarily activated by cytokines and environmental stress 98-100. Functional outcomes were different according to the specific JNK activation. In normal cells, JNKs can phosphorylate and regulate transcription factors such as c-Jun N-terminal Kinase 1 (JNK1), activating transcription factor 2 (ATF2), ETS domain-containing protein Elk-1 (ELK-1), tumor protein 53 (p53), recombinant c-Myc binding protein (MYCBP), and other non-transcription factors such as the Bcl family, in response to various extracellular stimuli 101-103. JNKs are considered to be either positive or negative regulators of cancer and are associated with cell survival, apoptosis, malignant transformation, and tumor development, depending on the cell type and lineage being studied 104. For instance, Recombinant thrombospondin 1(THBS1) derived from oral squamous cell carcinoma exosomes participates in the polarization of macrophages to an M1-like phenotype through p38, Akt, and SAPK/JNK signaling in the early phase 105. Additionally, dioscin elicits anti-tumor immunity by inhibiting M2 macrophage polarization through the JNK and STAT3 pathways in lung cancer 106. Currently, there is little research on nanotherapy targeting the JNK signaling pathway in TAMs. Most nanodrugs are used as carriers to encapsulate small-molecule drugs to modulate target genes. For instance, the sphingosine 1-phosphate receptor 1 (S1PR1) antagonist Ex26, modified by Zhou* et al*., was used to deliver the oncogene c-Myc inhibitor JQ1 via S1PR1 to TAMs. This approach effectively inhibits tumor-derived exosome-induced M2 polarization through the JNK signaling pathway 107. We believe that targeting the JNK signaling pathway in TAMs represents a promising therapeutic strategy for reprogramming TAMs, and further exploration is needed to achieve a reliable and strong therapeutic effect.

#### PI3K pathway

PI3K is a member of the lipid kinase family 108 and is first discovered 30 years ago 109. It has been classified into three types (I, II, III) in mammals, with particular attention given to class I PI3K in cancer research 110-112. PI3K activation primarily involves binding to substrates near the inner side of the plasma membrane 113, 114. Studies have shown that the PI3K/Akt signaling pathway plays an important role in macrophage activation and gene expression. The impact of Akt kinase on macrophage polarization varies, as Akt1 gene knockout (Akt1-/-) leads to M1 polarization, whereas Akt2-/- results in M2 polarization 115, 116. Furthermore, exosome-derived miRNAs from M2 macrophages can inhibit glioblastoma cell migration and invasion through the PI3K/AKT/mTOR signaling pathway 117.

To achieve dual targeting of TAMs and tumor cells in pancreatic cancer, Li* et al.* used a TME-responsive micellar system to co-load gemcitabine (GEM) and the PI3K inhibitor wortmannin (Wtmn). Specifically, GEM was covalently linked to dendritic poly-lysine DGL (GD) nanoparticles, which were then connected via a cathepsin B (CTSB) substrate peptide to polycaprolactone-polyethylene glycol micelles encapsu-lating Wtmn (PP/Wtmn) to form raspberry-like GD@PP/Wtmn micelles. Inhibiting the PI3K pathway repolarizes M2-like TAMs into M1-like TAMs, activating the anti-tumor immune response and synergizing with GEM to further suppress tumor growth 118*.* Li* et al.* developed a nanomicelle targeting M2 TAMs, which is capable of co-delivering the PI3K-γ inhibitor NVP-BEZ 235 and CSF-1R-siRNA for specific TAMs reprogramming and activation of anti-tumor immune responses. The formulated nanomicelles showed improved targeting efficiency towards M2 TAMs both in vitro and in vivo. Additionally, compared to single pathway blockade, the dual blockade of PI3K-γ and CSF-1R has demonstrated enhanced remodeling effects on TAMs by reducing the number of M2 TAMs and increasing that of M1 TAMs, further suppressing the infiltration of MDSCs 119. Furthermore,* Li et al.* synthesized porous hollow iron oxide nanoparticles (PHNPs) for loading a PI3K-γ small-molecule inhibitor (3-methyladenine, 3-MA) and further modified them with mannose for TAM targeting. The delivery system PHNPs@DPA-S-S-BSA-MA@3-MA exhibited excellent specificity. The combined action of PHNPs and 3-MA activates the inflammatory factor NF-κB p65 in macro-phages, synergistically switching TAMs into pro-inflammatory M1-type macrophages 120. Therefore, although there are few nanodrugs targeting this pathway, combining a nanosystem with a PI3K inhibitor is a promising approach to overcome the short-comings of traditional drugs.

#### Notch pathway

The Notch signaling pathway consists of four main components: Notch receptors, Notch ligands, CSL (CBF-1, Suppressor of hairless, Lag) DNA-binding proteins, and downstream target genes. The Notch signaling pathway regulates many aspects of tumor initiation and progression 121, in which the DNA-binding protein RBP-J/CBF1 mediates the main transcriptional signal of Notch receptors 122-124, activating macrophages to exhibit stronger tumor-suppressive effects 125, 126. Notch cleaves its transcriptionally active intracellular domain through a three-step proteolytic cleavage, releasing it to bind with the transcription factor CSL and regulate downstream gene expression. Deletion of CSL in monocytic lineages blocks TAMs differentiation and TAM-mediated immunosuppressive functions 127. Notch is highly expressed in drug-resistant breast cancer, leading to the differentiation of TAMs into an M2 phenotype and acquiring drug resistance 128. To date, there are no available studies regarding the application of nanodrugs that directly target the Notch signaling pathway. However, Scheurlen* et al*. proposed that integrating Notch-activating ligands (such as Dll1) and/or Notch1 overexpression plasmids into mannose-modified nanoparticles could selectively regulate the Notch signaling pathway in TAMs 129. Further research into nanodrugs that modulate Notch signaling in TAMs could pave the way for innovative cancer treatments.

## Conclusions and future perspectives

Macrophages possess high plasticity and show abundant infiltration in various pathological tissues, such as tumors, inflammation, rheumatoid arthritis, and parasitic infections. These are closely associated with the occurrence, development, and complications of the disease. Under the influence of a complex TME, macrophages infiltrating the tumor, which we defined as TAMs, undergo M2-like polarization and pro-mote tumor progression. To exert a cytotoxic effect on tumors, it is important to target TAMs, as well as a mainstream immunotherapy strategy for tumor treatment. With the development of nanodrugs, the efficacy of traditional targeted TAMs drugs can be improved, particularly synergizing with other treatments. However, the biggest challenge for nanodrugs lies in their future clinical translation. Although many nanodrugs have shown promising results in preclinical studies, there are still many issues regarding their clinical efficacy and safety that need to be addressed. Since nanoparticles can accumulate in non-target tissues to bring potential toxicity or trigger immune responses, resulting in insufficient treatment efficacy and severe adverse effects, the biocompatibility and safety of nanodrugs are major concerns. Biodistribution and targeting efficiency are also critical issues, which include non-specific uptake by non-target cells and limited penetration into dense tumor tissues. The complex synthesis and quality control of nanoparticles make large-scale production to be challenging and costly, due to the ensuring consistency in particle size, shape, and surface properties. For regulatory and clinical trial challenges, they include the lack of established guidelines for safety and efficacy evaluation, as well as the complexity of trial design to account for the unique pharmacokinetics of nanoparticles. Additionally, the high cost of development and production may limit the accessibility of nanodrugs, especially in low-resource settings. In summary, although the path to clinical translation is fraught with challenges, nanodrugs offer significant potential for enhancing cancer immunotherapy by targeting TAMs. Addressing these challenges will require interdisciplinary efforts, including advances in materials science, pharmacology, and clinical research. Once overcoming these hurdles, nanodrugs have the potential to revolutionize cancer treatment and improve patient outcomes.

## Figures and Tables

**Table 1:** Nanodrugs targeting the signaling pathways in TAMs to promote M1 polarization.

Pathways	Nanodrugs	Biological effect	Ref.
NF-κB	Artemisia-derived nanovesicles (ADNVs)	Carrying plant-derived mitochondrial DNA to activate cGAS-STING/NF-κB pathway	58
	Nanomicelle dissolving microneedle (DMN)	Loading cIAP inhibitor LCL-161 and R848 agonist to activate NF-κB pathway	60
	Silica nanoparticles (SNs)	Activating the mechanosensitive protein Piezo1, leading to calcium ion influx, NF-κB pathway activation	61
	Ginsenoside nanoparticles (GDNPs)	Containing ceramide lipids and proteins from ginseng to activate NF-κB pathway	62
	Poly(I:C) functionalized ferumoxytol (FMT) nanoparticles (FP-NPs)	Combining FMT and poly(I:C) to activate NF-κB pathway	63
	Mannose-modified PLGA (MAN-PLGA-N)	Using precision nanoparticle-based ROS photogeneration to activate NF-κB pathway	64
HIF-1α	Sophorolipid-associated membrane-biomimetic choline phosphate-poly(lactic-co-glycolic) acid hybrid nanoparticle (SDPN)	Delivering luteolin and silibinin act synergistically on STAT3 and HIF-1α pathway	69
	CL4H6 lipid nanoparticle (CL4H6-LNP)	Delivering HIF-1α siRNA to silence HIF-1α pathwaty	70
ROS	Ovalbumin- Fe-gallic acid (OFG) nanoparticles	Emulating the NOX2 enzyme's sequential ROS generation process	81
	Nanoliposome C6-Ceramide (LipC6)	LipC6 injection significantly regulate IRF1, 2, 3, 6 and activate ROS production	82
	Fe3O4 nanoparticle	Inducing ferroptosis by activating the generation of ROS	83
	Platinum(IV)/chloroquine/ perfluorohexane nanoparticle - anti-PD-L1 peptide (Pt(IV)/CQ/PFH NPs-DPPA-1)	Reprogramming the metabolic pathway and activating ROS	84
STAT	Carbon nanodot@mesoporous silica nanoparticles (CD@MSNs)	Stimulating NK cells to secrete IFN-γ that activate STAT1	94
	Paclitaxel-loaded ginsenoside Rg3 liposomes (Rg3-PTX-LPs)	Inhibiting IL-6/STAT3/p-STAT3 pathway activation	95
	Two-dimensional carbon-based nanomaterials-graphdiyne oxide nanosheets (GDYO)	Interacting with an intracellular protein corona consisting of STAT3 to inhibit STAT3 activation	96
	DPPA-1 peptide@oleanolic acid (DPAM@OA)	Elevating the expressions of VACM-1 and ICAM-1 and reducing the level of p-STAT3	97
JNK	Sphingosine 1-phosphate receptor 1 antagonist Ex 26/ JQ1 (Ex 26-CSOPOSA/JQ1)	Delivering the oncogene c-Myc inhibitor JQ1 to inhibiting JNK pathway	107
PI3K	Gemcitabine-dendritic poly-lysine@ polycaprolactone-polyethylene glycol micelles/Wtmn (GD@PP/Wtmn)	Loading PI3K inhibitor wortmannin(Wtmn) to TAMs	118
	M2pep-mixed micelle/BEZ 235/siRNA (M2pep-MM/BEZ/siRNA)	Encapsulating BEZ 235 and CSF-1R siRNA to block of PI3k-γ and CSF-1R	119
	Porous hollow iron oxide nanoparticles@DPA-S-S-bovine serum albumin-mannose@3-methyladenine (PHNPs@DPA-S-S-BSA-MA@3-MA)	loading a P13K-γ small molecule inhibitor and further modified by mannose to target TAMs	120
